# COVID-19 Vaccine Side Effects among Early-Vaccinated Healthcare Workers: Correspondence

**DOI:** 10.4314/ejhs.v32i6.24

**Published:** 2022-11

**Authors:** Won Sriwijitalai, Viroj Wiwanitkit

**Affiliations:** 1 Private Academic Consultant, Dimapur, India; 2 Honorary professor, Dr DY Patil University, Pune, Indi

**Dear Editor,**

we would like to share ideas on the publication “Prevalence of COVID-19 Vaccine Side Effects among Early-Vaccinated Healthcare Workers in Eastern Ethiopia (1)”. Jarso et al. reported that among recipients of the first dose of ChAdOx1 nCoV-19 at Adama Hspital Medical College, vaccine side effects were prevalent and discovered to be well-tolerated. The majority of the side effects were mild to moderate. To find rare adverse events, more side-effect profiles need to be researched and shared. Despite the likelihood of unfavorable side effects from immunization, clinical medicine emphasizes the clinical impact of the COVID-19 vaccine on other systems. The clinical issue identified in the recent study may or may not have been brought on by a harmful vaccination side effect. The recipient's physical or immunological condition prior to vaccination is not well understood. Additionally, the vaccine recipient can experience a different illness, which might be misinterpreted as a side effect (2). Another prospective analysis of the COVID-19 vaccine's effects on people with thorough clinical histories is required. A thorough laboratory analysis is needed after an incidence has been reported to rule out any other possibilities.

## References

[1] Jarso G, Gebi W, Abdo M, Lemma M, Abebe E, Lemessa B, Deressa BT (2022). Prevalence of COVID-19 Vaccine Side Effects among Early-Vaccinated Healthcare Workers in Eastern Ethiopia. Ethiop J Health Sci.

[2] Kebayoon A, Wiwanitkit V (2021). Dengue after COVID-19 vaccination: possible and might be missed. Clin Appl Thromb Hemost.

